# Bacterial clinical infectious diseases ontology (BCIDO) dataset

**DOI:** 10.1016/j.dib.2016.07.018

**Published:** 2016-07-16

**Authors:** Claire L. Gordon, Chunhua Weng

**Affiliations:** a Department of Medicine, Columbia University Medical Center, 630 West 168th Street, New York, USA; b Department of Biomedical Informatics, Columbia University Medical Center, 622 West 168th Street, New York, NY 10032, USA; c Department of Medicine, University of Melbourne, Melbourne, VIC 3010, Australia

**Keywords:** Biomedical informatics, Ontology, Infectious diseases

## Abstract

This article describes the Bacterial Infectious Diseases Ontology (BCIDO) dataset related to research published in http:dx.doi.org/ 10.1016/j.jbi.2015.07.014 [1], and contains the Protégé OWL files required to run BCIDO in the Protégé environment. BCIDO contains 1719 classes and 39 object properties.

**Specifications Table**

**Table:** 

Subject area	*Medicine, Biomedical informatics*
More specific subject area	*Bacterial Clinical Infectious Diseases Ontology*
Type of data	*Figure, Protégé source files*
How data was acquired	*Ontology was developed by Claire L. Gordon and includes imports from the OBO Foundry, Infectious Disease Ontology, Foundational Model of Anatomy and NCBI Taxon. Classes were mapped to Unified Medial Language System (UMLS) concept unique identifiers (CUIs) where possible.*
Data format	*Formatted*
Experimental factors	*Preparation of BCIDO was as follows: (1) determination of the domain and scope of the ontology; (2) review of the literature and related ontologies to evaluate them for reuse; (3) knowledge representation; and (4) evaluation.*
Experimental features	*BCIDO data is represented in the Web Ontology Language (OWL) as a single hierarchical structure using the Protégé-OWL editor Version 4.1 (*http://protege.stanford.edu*). Clinical ID concepts and antimicrobials in BCIDO were mapped to the reference resource Unified Medical Language System concept unique identifiers where possible. Bacterial terms were imported from the National Center for Biotechnology Information Organismal Classification (NCBITaxon). Anatomical terms were imported from The Foundational Model of Anatomy (FMA) (*http://sig.biostr.washington.edu/projects/fm/index.html*).*
Data source location	*n/a*
Data accessibility	*Data is submitted with this article*

**Value of the data**

• BCIDO may be useful for improving interoperability of antibiotic decision support systems.
• BCIDO may be used as a knowledge representation framework for clinical infectious disease data.
• BCIDO can be compared with other infectious disease ontologies to obtain further insight.
• BCIDO may be reused for designing an antibiotic decision support system.

## 1 Data

**Fig. 1:**
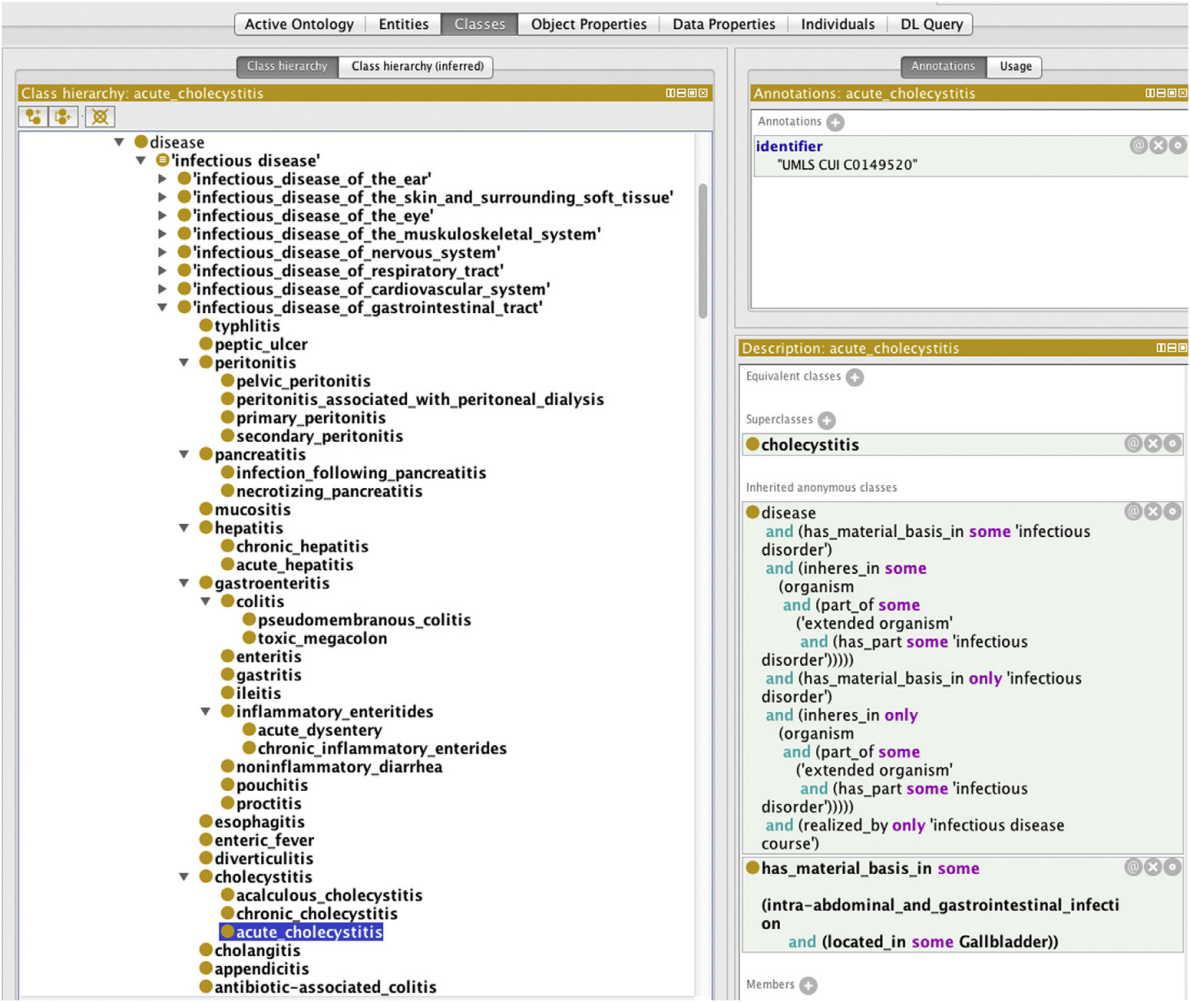
Bacterial infectious disease ontology (BCIDO) domain class of infectious disease showing ”acute cholecystitis” as an example.

BCIDO is represented in the Web Ontology Language (OWL) as a single hierarchical structure using the Protégé-OWL editor Version 4.1 (http://protege.stanford.edu). Fig. 1 shows the infectious disease domain class hierarchy. BCIDO contains 1719 classes, 39 object properties, 18 individuals, 2247 subsumption relations (SubClassOf axioms), 2770 logical axioms, 86 EquivalentClasses axioms and 350 DisjointClasses axioms.

## 2 Experimental design, materials and methods

The design of BCIDO has been described previously [2]. The data contained in BCIDO broadly covers the domain of clinical infectious diseases, and integrates the three major determinants of clinical infectious disease management (e.g. the infectious disease, the causative bacteria and the treating antibiotic). The accuracy and coverage of the data in BCIDO was assessed using a semi-automated method, as described [1]. To open BCIDO in Protégé Version 4.1, an open source collaborative ontology editing environment that is downloadable from Stanford University (http://protege.stanford.edu), open the file “BCIDO FINAL DIB.owl”. The required imported files are also contained within the “BCIDO” folder and will import automatically. Click “No” if asked to resolve missing imports.
