# Supplementary material for: Bacterial clinical infectious diseases ontology (BCIDO) dataset
Source: Data Brief. 2016 Jul 16;8:881–4. doi: 10.1016/j.dib.2016.07.018 (PMC4961784; doi:10.1016/j.dib.2016.07.018)
Supplement: Supplementary file 2 — Supplementary material [file mmc2.docx]

*Data article*

**Title: *Bacterial Clinical Infectious Diseases Ontology (BCIDO) Dataset***

**Authors: Claire L. Gordon^a,b,c^, Chunhua Weng^b^**

**Affiliations: *^a^ Department of Medicine, Columbia University Medical Center, 630 West 168^th^ Street, New York, USA***

***^b^ Department of Biomedical Informatics, Columbia University Medical Center, 622 West 168th Street, New York, NY 10032, USA***

***^c^ Department of Medicine, University of Melbourne, Melbourne VIC 3010, Australia***

**Contact email:** [cw2384@cumc.columbia.edu](mailto:cw2384@cumc.columbia.edu)

**Abstract**

This article describes the Bacterial Infectious Diseases Ontology (BCIDO) dataset related to research published in DOI 10.1016/j.jbi.2015.07.014 [1], and contains the Protégé OWL files required to run BCIDO in the Protégé environment. BCIDO contains 1719 classes and 39 object properties.

**Specifications Table**

| Subject area | *Medicine, Biomedical informatics* |
| --- | --- |
| More specific subject area | *Bacterial Clinical Infectious Diseases Ontology* |
| Type of data | *Figure, Protégé source files* |
| How data was acquired | *Ontology was developed by Claire L. Gordon and includes imports from the OBO Foundry, Infectious Disease Ontology, Foundational Model of Anatomy and NCBI Taxon. Classes were mapped to Unified Medial Language System (UMLS) concept unique identifiers (CUIs) where possible.* |
| Data format | *Formatted* |
| Experimental factors | *Preparation of BCIDO was as follows: (1) determination of the domain and scope of the ontology; (2) review of the literature and related ontologies to evaluate them for reuse; (3) knowledge representation; and (4) evaluation.* |
| Experimental features | *BCIDO data is represented in the Web Ontology Language (OWL) as a single hierarchical structure using the Protégé-OWL editor Version 4.1 ([http://protege.stanford.edu](http://protege.stanford.edu/)).* *Clinical ID concepts and antimicrobials in BCIDO were mapped to the reference resource Unified Medical Language System concept unique identifiers where possible. Bacterial terms were imported from the National Center for Biotechnology Information Organismal Classification (NCBITaxon). Anatomical terms were imported from The Foundational Model of Anatomy (FMA)(*[*http://sig.biostr.washington.edu/projects/fm/index.html*](http://sig.biostr.washington.edu/projects/fm/index.html)*).* |
| Data source location | *n/a* |
| Data accessibility | *Data is submitted with this article* |

**Value of the data**

- BCIDO may be useful for improving interoperability of antibiotic decision support systems
- BCIDO may be used as a knowledge representation framework for clinical infectious disease data
- BCIDO can be compared with other infectious disease ontologies to obtain further insight
- BCIDO may be reused for designing an antibiotic decision support system

**Data**

BCIDO is represented in the Web Ontology Language (OWL) as a single hierarchical structure using the Protégé-OWL editor Version 4.1 ([http://protege.stanford.edu](http://protege.stanford.edu/)). Figure 1 shows the infectious disease domain class hierarchy. BCIDO contains 1719 classes, 39 object properties, 18 individuals, 2247 subsumption relations (SubClassOf axioms), 2770 logical axioms, 86 EquivalentClasses axioms and 350 DisjointClasses axioms.

**Experimental Design, Materials and Methods**

The design of BCIDO has been described previously (2). The data contained in BCIDO broadly covers the domain of clinical infectious diseases, and integrates the three major determinants of clinical infectious disease management (e.g. the infectious disease, the causative bacteria and the treating antibiotic). The accuracy and coverage of the data in BCIDO was assessed using a semi-automated method, as described (1). To open BCIDO in Protégé Version 4.1, an open source collaborative ontology editing environment that is downloadable from Stanford University (<http://protege.stanford.edu>), open the file “BCIDO FINAL DIB.owl”. The required imported files are also contained within the “BCIDO” folder and will import automatically. Click “No” if asked to resolve missing imports.

**Acknowledgements**

We are very grateful to Lindsay Cowell and Albert Goldfain for assistance with designing BCIDO. This work was conducted using the Protégé resource, which is supported by grant GM10331601 from the National Institute of General Medical Sciences of the United States National Institutes of Health. This research was partially supported by National Library of Medicine grant R01LM009886 to Dr. Weng. Dr. Gordon is supported by a Hutchins Family Fellowship, Fulbright Postgraduate Scholarship and Australian American Association Fellowship. The article’s contents are solely the responsibility of the authors and do not necessarily represent the official view of NIH.

**References**

(1) Gordon, C.L., and C. Weng. 2015. Combining expert knowledge and knowledge automatically acquired from electronic data sources for continued ontology evaluation and improvement. *J Biomed Inform* 2015 Jul 23. pii: S1532-0464(15)00154-9. doi: 10.1016/j.jbi.2015.07.014. [Epub ahead of print]

(2) Gordon CL, Pouch S, Cowell LG, Boland MR, Platt HL, Goldfain A, et al. Design and evaluation of a bacterial clinical infectious diseases ontology. AMIA Annual Symposium proceedings / AMIA Symposium AMIA Symposium. 2013;2013:502-11.
