# Supplementary material for: Bacterial clinical infectious diseases ontology (BCIDO) dataset
Source: Data Brief. 2016 Jul 16;8:881–4. doi: 10.1016/j.dib.2016.07.018 (PMC4961784; doi:10.1016/j.dib.2016.07.018)
Supplement: Supplementary file 3 — Supplementary material [file mmc3.zip › BCIDO FINAL/Neorick.html]

OntoFox


HomeIntroductionTutorialFAQsReferencesDownloadLinksContactAcknowledgeNews

Retrieving Results

**Finished retrieving process. Please download the output file.**

Your input file should be located at http://ontofox.hegroup.org/userfiles/bcgoquz4.txt and your output file should be located at http://ontofox.hegroup.org/userfiles/bcgoquz4.owl. Please includes these two links in your email if you need our assistance.

These files will be destroyed at 3:00 AM EST (New York time). If you wish to destroy these files now, please click here.

OntoFox Survey: your feedback on OntoFox is welcome and important for us to improvie this service. This survey contains 16 questions and will take approximately 5 minutes. Thank you!

Show SPARQL queries used in this page

```
prefix p_2: <http://>
prefix p_3: <http://purl.obolibrary.org/obo/>
prefix p_1: <http://purl.obolibrary.org/obo/merged/>
SELECT DISTINCT ?s
FROM p_1:NCBITaxon
WHERE {
	?s ?p ?o.
	FILTER (?s in (p_2:null, p_3:NCBITaxon_33993))
}


====================================================================

prefix p_3: <http://purl.obolibrary.org/obo/>
prefix p_2: <http://purl.obolibrary.org/obo/merged/>
prefix p_1: <http://www.w3.org/1999/02/22-rdf-syntax-ns#>
CONSTRUCT {
?s p_1:type ?o
}
FROM p_2:NCBITaxon
WHERE {
?s p_1:type ?o.
FILTER (?s in (p_3:NCBITaxon_33993))
}


====================================================================

prefix p_3: <http://purl.obolibrary.org/obo/>
prefix p_2: <http://purl.obolibrary.org/obo/merged/>
prefix p_1: <http://www.w3.org/2000/01/rdf-schema#>
CONSTRUCT {
?s p_1:subClassOf ?o
}
FROM p_2:NCBITaxon
WHERE {
?s p_1:subClassOf ?o.
FILTER (?s in (p_3:NCBITaxon_33993))
}


====================================================================

prefix p_3: <http://purl.obolibrary.org/obo/>
prefix p_2: <http://purl.obolibrary.org/obo/merged/>
prefix p_1: <http://www.w3.org/2000/01/rdf-schema#>
CONSTRUCT {
?s p_1:subPropertyOf ?o
}
FROM p_2:NCBITaxon
WHERE {
?s p_1:subPropertyOf ?o.
FILTER (?s in (p_3:NCBITaxon_33993))
}


====================================================================
```

|  |  |
| --- | --- |
| He Group  University of Michigan Medical School  Ann Arbor, MI 48109 |  |
