# Supplementary figures and images for: Bacterial clinical infectious diseases ontology (BCIDO) dataset
Source: Data Brief. 2016 Jul 16;8:881–4. doi: 10.1016/j.dib.2016.07.018 (PMC4961784; doi:10.1016/j.dib.2016.07.018)

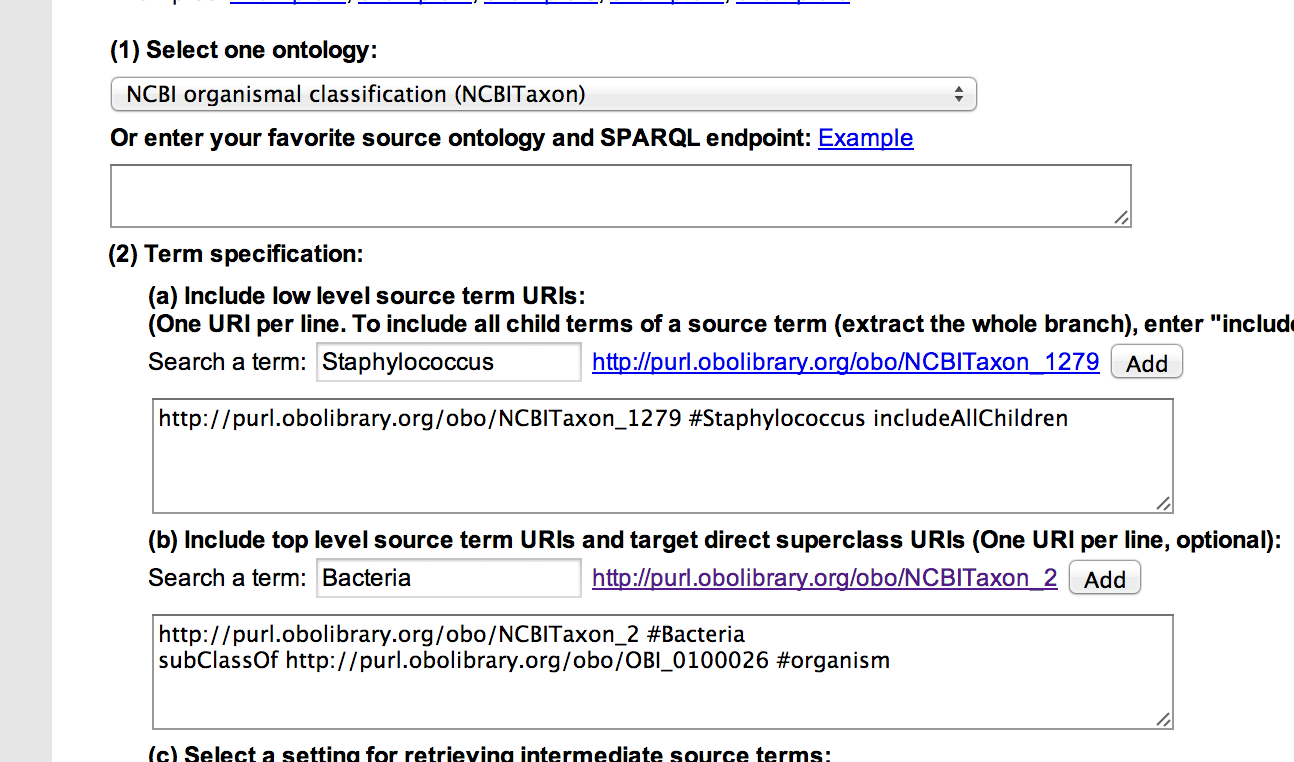

Supplement: Supplementary file 3 — Supplementary material [file mmc3.zip › BCIDO FINAL/Screen Shot 2013-05-16 at 3.55.16 PM.png]
